# Perinatal Survival and Predictors of Mortality among Mothers with Hypertensive Disorders of Pregnancy at Antenatal care Clinics in Gamo Zone Public Hospitals

**DOI:** 10.4314/ejhs.v30i6.2

**Published:** 2020-11

**Authors:** Samuel Dessu Sifer, Fikre Bojola, Zinabu Dawit, Habtamu Samuel, Mulugeta Dalbo

**Affiliations:** 1 Department of Public Health, College of Medicine and Health Sciences, Wolkite University, Wolkite, Southern Ethiopia; 2 Department of Nursing, Arba Minch Health Science College, Arba Minch, Southern Ethiopia; 3 Department of Public health, Arba Minch Health Science College, Arba Minch, Southern Ethiopia; 4 Department of Midwifery, Arba Minch Health Science College, Arba Minch, Southern Ethiopia

**Keywords:** Incidence, Predictors, Perinatal mortality, Antenatal care

## Abstract

**Background:**

Pregnancy induced hypertension represents a significant public health problem throughout the world, which may complicate 0.5%–10% of all pregnancies. It is the leading cause of maternal as well perinatal mortality and morbidity worldwide. Pregnancy induced hypertension is a multisystem disorder unique to pregnancy and results in high perinatal mortality. The objective of this study was to determine the survival status, incidence and predictors of perinatal mortality among mothers with pregnancy induced hypertension at antenatal clinics of Gamo Zone public hospitals.

**Methods:**

Facility-based retrospective cohort study was conducted among selected 576(192 exposed and 384 unexposed) antenatal care attendants' record at Gamo Zone public hospitals from 1^st^ January 2018 to 31^st^ December 2018. Data were entered into Epi data version 3.02 and exported to SPSS V 25 for analysis. Kaplan Meier survival curve together with log rank test was fitted to test the survival time. Statistical significance was declared at P-value ≤0.05 using cox proportional hazard model.

**Result:**

The incidence of perinatal mortality was 124/1000 births. The cumulative proportion of surviving at the end of 4^th^, 8^th^, 12^th^ and 16^th^ weeks of follow-up among the exposed groups was 96.9%, 93.5%, 82.1% and 61.6% respectively whereas it was 99.5%, 98.9% and 98.5% at the end of 4^th^, 8^th^ and 12^th^ weeks of follow-up for the non-exposed groups respectively. Parity of ≥5(AHR: 6.3; 95%CI: 1.36,10.55), mothers who delivered at <34 weeks of gestation(AHR:7.8; 95%CI: 2.6,23.1), being preterm(AHR:6; 95%CI: 5.3,19.2), perinatal birth weight ≤2500gm(AHR:6.1; 95&CI: 1.01,37.9), vaginal deliveryn(AHR:2.7; 95%CI:1.13,6.84), maternal highest systolic blood pressure level ≥160mmHg (AHR: 2.3; 95%CI: 1.02,5.55) and prepartum onset of pregnancy induced hypertension (AHR: 6; 95%CI: 5.3,19.2) were statistically significant in multivariable analysis.

**Conclusion:**

The risk of perinatal mortality was high among the mothers with pregnancy induced hypertension compared to those of pregnancy induced hypertension free mother,s and the perinatal mortality rate was high. High parity, low gestational age, low number of antenatal care visits, low birth weight, vaginal delivery, antepartum onset of pregnancy induced hypertension and highest maternal systolic blood pressure level were the independent predictors of perinatal mortality.

## Introduction

Pregnancy Induced Hypertension (PIH) is a term used to define a wide spectrum of women who may have only mild elevations in blood pressure (BP) or severe Hypertension (HTN) with various organ dysfunctions (1). It complicates nearly 0.5%–10% of all pregnancies, which is the leading causes of maternal and perinatal mortality and morbidity worldwide (2).

Preeclampsia and eclampsia are the most common complications of pregnancy induced hypertension; and are the recognized causes of maternal and newborn morbidity and mortality in low and middle income countries. Preeclampsia can be present as HELLP syndrome (hemolysis, elevated liver enzymes and low platelet count) or eclampsia that is occurrence of convulsions that cannot be attributed to other etiologic factors (1).

According to the 2017 world preeclampsia day report, as a leading cause of maternal mortality, preeclampsia and related pregnancy induced hypertension claim the lives of nearly 76,000 mothers and 500,000 babies worldwide every year (3). Magnesium sulfate is the drug recommended for the prevention of complications (seizure) as part of comprehensive management of pregnancy induced hypertension (4).

According to the World Health Organization (WHO), multi-country survey, there was about three-fold increased risk of perinatal death in women with preeclampsia and five-fold increased risk of perinatal death in women with eclampsia compared to women with no preeclampsia or eclampsia (2). The majority of perinatal deaths due to complications of PIH have occurred in the low income and middle income countries.

In Ethiopia, the complications of PIH are major causes of maternal and perinatal morbidities and mortalities. PIH is one of the significant public health problems throughout the world, and preeclampsia is the most common of these problems (5).

Ethiopia is one of the countries in sub-Saharan Africa having high perinatal mortality. A study conducted on trend of perinatal mortality rate revealed that a rate of 90 and 40 per 1000 birth in hospital and community-based settings respectively (5), and according to EDHS 2016, it was 33 per 1000 pregnancies (6). In addition, the progress in reduction in rate of still birth was too much poor. The Ethiopian Demographic and Health Survey (EDHS) 2000, 2011 and 2016 revealed that the rate of still birth was 10.4, 16.9 and 11 per 1000 births respectively (6,7,8). Similarly, a study conducted in the southern part of Ethiopia reported that there was a considerable association between perinatal deaths and low birth weight (9). Obstetric related factors and pregnancy induced hypertension related factors were the most common factors affecting the perinatal survival status. Therefore, the objective of this study was to determine the survival time, incidence and predictors of perinatal mortality among mothers with pregnancy induced hypertension at antenatal care clinics of Gamo Zone public hospitals from January 01, 2018 to December 31, 2018.

## Methods

**Study design, area and period**: A retrospective cohort study was conducted at Gamo Zone public hospitals from 1^st^ January, 2018 to 31^st^ December, 2018. Gamo Zone is one of the zones found in South Nations Nationalities and People's Regional State (SNNPRS). The zone has 3 hospitals (Arba Minch General Hospital, Kemba Primary Hospital and Chencha District Hospital).

**Population**: All records of Antenatal Care (ANC) attendant pregnant mothers who had PIH at Gamo Zone public hospitals were considered as the source population, and the study population was all the selected records of antenatal care attendant pregnant mothers who had PIH at Gamo Zone public hospitals. Records of antenatal care attendant pregnant mothers who had PIH at Gamo Zone public hospitals were considered as exposed groups, and records of pregnancy induced hypertension free antenatal care attendant pregnant mothers were considered as unexposed groups.

**Selection of study subjects**: All babies born from mothers who had ANC visit at Gamo Zone public hospitals from 1^st^ January, 2018 to 31^st^ December, 2018 were included in the study and babies born before 28 weeks of gestation, babies born from mothers who had chronic hypertension before pregnancy and incomplete records were excluded from the study. The diagnosis of PIH was made by the physicians and midwives working in the unit. It was an open cohort, and exposed subjects were entered into the cohort at the time on the diagnosis of pregnancy induced hypertension and unexposed one were entered at any time of the follow-up period. In addition, subjects exited from the cohort when discharged as cured at the end of treatment declared or died.

**Sample size determination and sampling procedure**: Arba Minch General Hospital and Chencha District Hospital were selected for this study using lottery method among hospitals at Gamo Zone. Within this hospital, there were 192 mothers who had PIH. Therefore, all the mothers who had PIH were taken as exposed group, and for each of the exposed group, two consecutive members of the unexposed group were taken for comparison. This yielded 576 (192 exposed and 384 unexposed groups) respondents.

**Study variables**: The dependent variable was time to perinatal death. The independent variables were categorized into obstetric related factors, PIH and related factors an (maternal age, parity, gestational age, number of ANC visits, labour onset, number of fetuses, mode of delivery and fetal birth weight) and PIH related factors (highest systolic blood pressure level, highest diastolic blood pressure level, type of PIH, onset of PIH, severity symptoms of PIH, type of anticonvulsant given, type of antihypertensive given and maternal hemoglobin level).

## Definition of Terms

**Perinatal death**: The death of a fetus/neonate in the perinatal period (from age of viability or twenty eight weeks of gestation in Ethiopian context to first six days after birth) (8). Pregnancy induced hypertension: A new hypertension that appears at 20 weeks or more gestational age of pregnancy with or without proteinuria, which includes gestational hypertension, preeclampsia and eclampsia (9).

**Data collection tool and Procedure**: Data were collected using a questionnaire prepared specific to this study using individual patient records including registers, monitoring cards and patient admission book and appropriate modification was made. In addition, the tool contains maternal age, obstetric related factors, pregnancy induced hypertension related factors and basic laboratory findings. The data were collected by four researchers (midwives) a supervised by two researchers (midwives) who were trained on basic emergency mother and neonatal care.

**Data processing and analysis**: Data were cleaned, edited, coded and entered into Epi data version 3.02 and exported to SPSS v 25 for analysis. Bivariate analysis was conducted to identify associations between dependent and independent variables. Hazard ratios, 95% CIs and P-values were used to assess the strength of association and statistical significance. Incidence of death was calculated. Variables which had p-value <0.25 level in the bivariate analysis were included in the final Cox-regression analysis. Variables that had a p-value of ≤0.05 in the multivariable analysis were considered as independent predictors of perinatal mortality in the final Cox-regression analysis.

**Ethical consideration**: Ethical clearance was obtained from Arba Minch College of Health Science Ethical Review Committee, and support letter was obtained prior to data collection. Permission letter was obtained from Gamo Zone Health Department. The administrators of Arba Minch General Hospital and Chencha District Hospital were informed about the objective of the study through a support letter. All the appropriate efforts were made to handle the confidentiality of the records.

## Results

This study included 576 (192 exposed and 384 unexposed) antenatal care attending pregnant mothers. The mothers gave a total of 595 babies (19 sets of twins and all the twins were from the unexposed groups) after 28 weeks of gestation. Specific to this study, there were 74 perinatal deaths resulting in a Perinatal Mortality Rate (PMR) of 124/1000 total births.

The distribution of PIH by type was mild preeclampsia 100(17.4%), sever preeclampsia 61(10.6%) and eclampsia 31(5.4%). However, the case fatality rate of preeclampsia (10%) was significantly lower than sever preeclampsia(63%) and eclampsia(64%). The majority of perinatal deaths 83.7% occurred among women with antepartum onset of PIH. Intra-partum and post-partum gestational age accounted for 32.4% and 8.1% respectively.

Among the total perinatal deaths in antepartum period, there were 59.5%, 24/74(32%) and 6/74(8.1%) perinatal mortalities at very preterm, preterm and term periods respectively. Among them, 93.2% were exposed to pregnancy induced hypertension. From those exposed, very preterm and preterm accounted for 60.9% and 30.4% respectively. The premature perinatal death was more pronounced in eclamptic mothers than in preeclamptic mothers (50.7% and 26.1% respectively) (Table 1).

**Table 1:** Obstetrics and pregnancy induced hypertension related characteristics of women attending ANC in Gamo Zone public hospitals, Southern Ethiopia, 2018 (n=576)

Variables	Category	Exposed(n=192)		Non exposed(n=384)	
		
		Died	Survived	Died	Survived
		n(%)	n(%)	n(%)	n(%)
**Parity**	Primigravida	11(5.72)	42(21.88)	1(0.3)	101(26.3)
	Multipara(I-IV)	44(22.9)	75(39.06)	2(0.5)	250(65.1)
	Grand Multipara(V+)	14(7.29)	6(3.1)	2(0.5)	28(7.3)
**Labor onset**	Spontaneous	54(28.1)	90(46.9)	5(1.3)	349(90.9)
	Induced	10(5.2)	24(12.5)	0(0)	19(4.9)
	Direct CS	5(2.6)	9(4.7)	0(0)	11(2.9)
**Mode of delivery**	Vaginal	51(26.6)	99(51.6)	5(1.3)	352(91.7)
	Cesarean section	18(9.4)	24(12.5)	0(0)	27(7.0)
**Gestational Age(week)**	<34	42(21.9)	15(7.8)	2(0.5)	44(11.5)
	34–36	21(10.9)	27(14.1)	3(0.8)	120(31.3)
	≥37	6(3.1)	81(42.2)	0(0)	215(56.0)
**Anticonvulsant provision**	Yes	54(28.1)	57(29.7)	0(0)	1(0.3)
	No	15(7.8)	66(34.4)	5(1.3)	378(98.4)
**Type of anticonvulsant**	MgSO4	48(42.9)	42(37.5)	0(0)	0(0)
**(n=112)**	Diazepam	6(5.4)	15(13.4)	0(0)	1(0.8)

**Figure 1:**
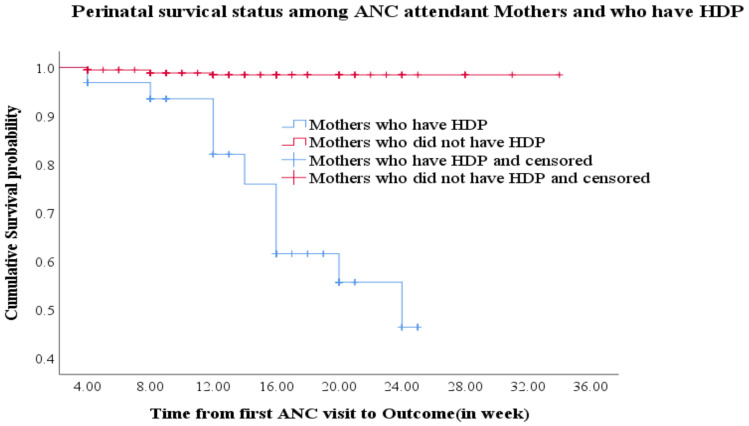
Kaplan-Meier survival estimate among mothers with pregnancy induced hypertension at ANC clinics of Gamo Zone public hospitals, Southern Ethiopia, 2018 (n=576).

**Perinatal Survival status**: A total of 576 perinates were followed for a minimum of four weeks and a maximum of 36 weeks with a median follow-up period of 14 weeks. Maximum of the respondents(18.6%) were followed for 20 weeks. In addition, 75(13%) of the women were followed for four weeks and 6(1%), 1(0.2%) and 1(0.2%) of the women were followed for 28, 31 and 34 weeks respectively. The cumulative proportion of surviving at the end of 4^th^, 8^th^, 12^th^ and 16^th^ weeks of follow-up among the exposed groups were 96.9%, 93.5%, 82.1% and 61.6% respectively whereas it was 99.5%, 98.9% and 98.5% at 4^th^, 8^th^ and 12^th^ weeks of follow-up for the unexposed group respectively. The overall mean survival time was 29.2[95%CI: 28.1, 30.4] weeks.(Figure 1).

**Figure 2:**
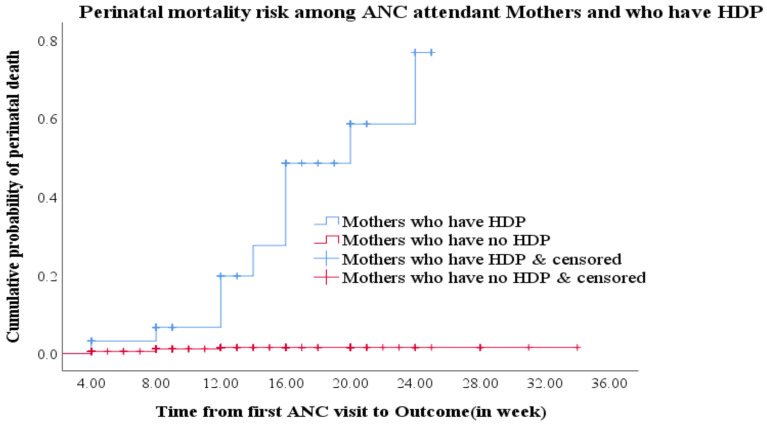
Difference in hazard of perinatal death between mothers with PIH and without PIH at ANC clinics of Gamo Zone public hospitals.

There was high number of perinatal mortality among the exposed group as compared to the unexposed group. As the gestational age increased, the perinatal mortality also increased among the mothers with PIH but as the gestational age increased, the survival probability increased among mothers who have no PIH (Figure 2).

**Comparison of survivor function between two or more group of perinates**: The log-rank test result indicated that survival pattern or time to perinatal mortality significantly varied among the covariates of the perinatal mortality predictors. The overall mean survival time for parity and birth weight was 29.2[95%CI: 28.1, 30.3] and 32.4[95%CI: 31.8, 33.0] respectively (Table 2).

**Table 2:** The log-rank test estimate and the mean survival time between the covariates of the perinates among both the exposed and the non exposed groups

Variable	Category	Mean survival time [95%CI]	Log rank test
**Parity**	Primigravida	28.7[27.6,29.9]	*x*^2^=35.9, p-value=0.0001
	I-IV	29.3[27.9,30.7]	
	≥V	15.8[14.4,17.4]	
**Gestational Age(week)**	≤34	19.6[17.2,22.2]	*x*^2^=117.3.9, p-value=0.0001
	35–36	24.3[23.1,25.7]	
	≥37	27.5[27.2,27.9]	
**Number of ANC visits**	1	19.3[14.7,24.4]	*x*^2^=55.8, p-value=0.0001
	2	17.2[16.6.19.2]	
	3	28.7[26.6,30.8]	
	≥4	28.2[27.3,29.2]	
**Birth weight(gm)**	<2500	28.7[26.05,31.39]	*x*^2^=29.3, p-value=0.0001
	≥2500	30.1[29.68,30.60]	
**Mode of delivery**	Vaginal	29.5[28.4,30.7]	*x*^2^=12.6, p-value=0.002
	CS	23.3[21.4,25.1]	
**Highest systolic blood**	<140	31.4[30.4,32.5]	*x*^2^=153.4, p-value=0.0001
**pressure(mmHg)**	141–159	17.3[15.4,19.3]	
	≥160	15.1[12.7,17.4]	
**Onset of pregnancy**	Pre partum	19.5[18.4,20.6]	*x*^2^=103.7, p-value=0.0001
**induced hypertension**	Intra partum	19.2[17.9,20.5]	
	Post-partum	18[16.0,19.9]	

**Comparison of survivor function between two or more group of perinates among the exposed groups**: The overall mean survival time for the variable parity was 19.93(95%CI:18.97,20.89). Similarly, the overall mean survival time for gestational age was 23.84 (95%CI: 22.95, 24.74). In addition, the overall mean survival time for number of antenatal care visits, birth weight, mode of delivery, highest systolic blood pressure and onset of pregnancy induced hypertension was 19.93(95%CI: 18.97,20.89), 22.59(95%CI: 21.64,23.55), 19.93(95%CI: 18.97,20.88), 19.93(95%CI: 18.97,20.89) and 19.93(95%CI: 18.97,20.89) respectively. The specific mean survival time for each category of the variable was mentioned in Table 3.

**Table 3:** The log-rank test result and the mean survival time among the covariates of the perinates delivered from the exposed groups

Variable	Category	Mean survival	Log rank test estimate
		time(95%CI	
**Parity**	Primigravida	21.45(20.11,22.79)	X^2^=38.45, P-value=0.0001
	Multipara(I-IV)	20.04(18.84,21.24)	
	Grand	12.55(10.99,14.12)	
	multipara(V+)		
**Gestational Age**	≤34	15.75(14.15,17.36)	X^2^=57.95, P-value=0.0001
	35–36	16.78(15.54,18.01)	
	≥37	23.84(22.95,24.74)	
**Number of ANC visits**	One	10.00(8.53,11.47)	X^2^=69.69, P-value=0.0001
	Two	11.08(10.09,12.07)	
	Three	17.53(16.34,18.72)	
	Four and above	21.75(20.81,22.71)	
**Birth weight (gram)**	<2500	15.37(13.26,17.48)	X^2^=20.68, P-value=0.0001
	≥2500	23(84(23.13,24.56)	
**Mode of delivery**	Vaginal	20.08(18.99,21.17)	X^2^=13.0, P-value=0.001
	Cesarean section	17.32(15.81,18.83)	
**Highest systolic blood**	<140	21.35(20.22,22.48)	X^2^=20.69, P-value=0.0001
**pressure(mmHg)**	141–159	17.74(15.88,19.59)	
	≥160	16.34(13.95,18.73)	
**Onset of pregnancy**	Pre partum	19.56(18.49,20.64)	X^2^=26.1, P-value=0.002
**induced hypertension**	Intra partum	19.28(17.97,20.58)	
	Post-partum	18.00(16.04,19.96)	

**Comparison of survivor function between two or more group of perinates among the non-exposed groups**: The mean survival time was different for each covariate of the unexposed group. The log rank test estimate was varied between the pooled estimate of the two groups and the unexposed group. The overall mean survival time for parity among the unexposed groups was 33.59 (95%CI: 33.25, 33.95). Similarly, the overall mean survival time for gestational age, number of antenatal care visits, birth weight and mode of delivery among the unexposed groups was 13.28 (95%CI: 12.67, 13.89), 13.28 (95%CI: 12.67, 13.89), 13.27 (95%CI: 12.66, 13.89) and 13.28 (95%CI: 12.67, 13.89) respectively (Table 4).

**Table 4:** The log-rank test estimate and the mean survival time among the covariates of the perinates delivered from the non-exposed groups

Variable	Category	Mean survival time(95%CI)	Log rank test estimate
**Parity**	Primigravida	30.74(30.22,31.25)	X^2^=7.338, P-value=0.026
	Multipara(I-IV)	33.75(33.41,34.09)	
	Grand multipara(V+)	19.04(17.76,20.32)	
**Gestational Age**	≤34	10.71(8.67,12.75)	X^2^=9.025, P-value=0.011
	35–36	13.34(12.21,14.47)	
	≥37	13.86(13.11,14.61)	
**Number of ANC visits**	One	6.51(4.92,8.10)	X^2^=23.985, P-value=0.0001
	Two	9.40(8.34,10.46)	
	Three	12.72(11.75,13.68)	
	Four and above	17.00(16.31,17.69)	
**Birth weight**	<2500	12.79(10.77,14.82)	X^2^=44.31, P-value=0.0001
	≥2500	13.33(12.69,13.97)	
**Mode of delivery**	Vaginal	13.16(12.54,13.79)	X^2^=38.01, P-value=0.0001
	Cesarean section	14.78(12.11,17.44)	

**Predictors of Perinatal mortality**: This study revealed that parity, gestational age, number of ANC visits, birth weight, mode of delivery, highest systolic blood pressure and onset of hypertensive disorders of pregnancy were the independent predictors of perinatal mortality.

The risk of perinatal mortality among women with parity of I-IV was twice higher compared to the primigravidas (AHR:2.1; 95%CI: 1.01,4.3). Mothers with a party of five and above have 6.3 times higher risk of perinatal mortality compared to the primi gravidas(AHR: 6.3; 95%CI: 1.36,10.55). Mothers who delivered before 34 weeks of gestation had 7.8 times higher risk of perinatal mortality compared to those of 37 weeks and above (AHR:7.8; 95%CI: 2.6,23.1). Preterm's (34–36weeks of gestation) had 6 times higher risk of mortality compared to those of terms(37 weeks and above) AHR:6; 95%CI: 5.3,19.2). Mothers who had one ANC visit had 8 times higher risk of perinatal mortality compared to those who had four and above visits (AHR: 8; 95%CI: 11.7,28.45). Similarly, mothers who had three and two ANC visits had 6.3 and 3.4 times higher risk of perinatal mortality as compared to four and above ANC visits (AHR; 6.3; 95%CI: 2.1, 18.7) and (AHR: 3.4; 95%CI: 2.1, 18.7) respectively.

Perinates with birth weights below 2500gm had 6.1 times higher risk of mortality compared to the counterparts who had 2500gm and above weight (AHR:6.1; 95&CI: 1.01,37.9). Mothers who delivered with a vaginal delivery had 2.7 times higher risk of perinatal mortality compared to those with who delivered with cesarean section (AHR: 2.7; 95%CI: 1.13, 6.84) (Table 5).

**Table 5:** Predictors of perinatal mortality among mothers with pregnancy induced hypertension at ANC clinics of Gamo Zone public hospitals, Southern Ethiopia(n=576)

Variables	Category	Total	Perinatal death (%)	CHR(95%CI)	AHR(95%CI)
**Residence**	Urban	458	12.7	1	
	Rural	118	18.6	1.4(0.9,2.3)*	
**Parity**	Primigravida	156	8.3	1	1
	I–IV	370	12.4	1.5(0.8,2.9)*	2.1(1.01,4.3)**
	≥V	50	32	6.3(2.9,13.3)*	3.7(1.36,10.55)**
**Gestational**	Very preterm(≤34)	103	42.7	25(10.7,58.8)*	7.8(2.6,23.1)***
**age(week)**	Preterm(34–36)	171	14.0	8.8(0.36,21.5)	6(5.3,19.2)***
	Term(≥37)	302	1.99	1	1
**Number of ANC**	One visit	74	16.2	10(5.1,21)*	8(11.7,28.45)***
**visits**	Two visit	88	9.1	3.4(1.5,7.6)*	6.3(2.1,18.7)***
	Three visits	142	14.1	2.1(1.5,7.6)*	3.4(1.6,6.8)***
	Four and above visits	272	12.5	1	1
**Birth weight(gram)**	<2500	70	18.6	6.4(2.9,14.1)*	6.1(1.01,37.9)**
	≥2500	457	2.6	1	
**Labour onset**	Spontaneous	498	11.8	0.86(0.34,2.15)	
	Induced	53	18.9	0.96(0.33,2.83)	
	Direct cesarean section	25	20	1	
**Mode of delivery**	Vaginal	507	11.0	1.9(1.1,3.24)*	2.7(1.13,6.84)**
	Cesarean section	69	26.1	1	1
**Number of fetus**	Single tone	557	12.7	1	
	Twin	19	15.8	1.1(0.36,3.63)	
**Maternal hemoglobin**	<10	54	11.1	0.79(0.34,1.84)	
**level(mg/dl)**	10–11.9	68	17.6	1.39(0.75,2.61)	
	>12	454	12.3	1	

Key note:

* p-value <0.25

** p-value <0.05

*** p-value <0.001

Mothers who had the highest systolic blood of PIH had 3 times higher risk of perinatal mortality compared to those of postpartum onset (AHR pressure 160mmHg and above have 2.3 times higher risk perinatal mortality compared to those who had 140mmHg and below (AHR:2.3; 95%CI: 1.02,5.55). Mothers who have a prepartum onset: 6; 95%CI: 5.3, 19.2) (Table 6).

**Table 6:** Pregnancy induced hypertension related predictors of perinatal mortality among mothers with PIH at ANC clinics of Gamo Zone public hospitals

Variables	Category	Total	Perinatal mortality (%)	CHR(95%CI)	AHR(95%CI)
**Highest systolic blood** **pressure(mmHg)**	<140	498	4.2	1	1
	141–159	37	59.5	8.6(4.9,14.9)*	
	≥160	41	75.6	11.6(6.7,20)*	2.3(1.02,5.55)**
**Highest Diastolic blood** **pressure(mmHg)**	<90	478	2.3	1	
	90–110	46	41.3	4.2(2.4,7.2)*	
	≥110	52	84.6	6(3.1,11.7)*	
**Onset of pregnancy induced** **hypertension**	Pre partum	158	39.2	5(0.01,0.27)*	3(0.004,0.239)***
	Intra partum	14	35.7	1.3(0.3,5.1)	0.77(0.15,3.8)
	Post-partum	20	10	1	1
**Type of pregnancy induced** **hypertension**	Mild preeclampsia	100	10	1	
	Sever preeclampsia	61	63.9	5.5(1.9,16.3)*	
	Eclampsia	31	64.5	4.6(18,118)*	
**Having severity symptoms**	Yes	74	55.4	3.2(1.9,2.9)*	
	No	118	27.9	1	
**Type of anticonvulsant** **provided**	MgSO4	48	87.5	1	
	Diazepam	64	25	10(6.4,18.5)*	

Key note:

* p-value <0.25

** p-value <0.05

*** p-value <0.001

## Discussion

Consistent with previous study findings, this study indicated that there was a high perinatal mortality among mothers with PIH (10,11,12,13). However, the perinatal mortality rate in this study was lower than the study report from Jimma, Ethiopia, and Hawassa University Teaching Hospitals (1,14), but which is greater than the study reports from Pakistan, Turkey and Addis Ababa (11,15,16). This might be due to the increased focus of the government and other stakeholders, variation in the implementation strategies across the globe and increased awareness and knowledge on PIH.

Specific to the objective of this study, the analysis demonstrated the independent predictors of perinatal mortality. In multivariable cox proportional hazard model, parity, gestational age, number of ANC visit, birth weight, mode of delivery, highest systolic blood pressure and onset of PIH were statistically significant.

In this study, an increase in the risk of perinatal mortality due to an increase in parity was observed. The finding of this study was consistent with other studies conducted in Hawassa University Teaching Hospital, which revealed that multiparas (I-IV) had 1.6 and grand multiparas had 2.8 times higher risk of perinatal mortality compared to primigravidas (14). Similarly, a study conducted at Derashe District revealed that grand multiparas had 4 times higher risk of perinatal mortality compared to the primigravidas (17). This might be due to the fact that multi-parity may predispose the mother to anemia, Diabetes Mellitus (DM), mal-presentation, abruption of placenta, placenta previa, post-partum hemorrhage due to uterine atony and uterine rupture (18,19,20).

The significantly increased risk of perinatal mortality among babies with low gestational age and low birth weight in this study is consistent with several other studies (14, 21, 22, 23). This finding shows a strong association of perinatal mortality among babies delivered from mothers having antepartum onset of PIH. More than two-thirds of the perinatal deaths (91%) in women with antepartum onset of PIH were either preterm or very preterm at birth. This might be due to the fact that PIH probably exposed several babies to premature delivery and related complications. Inconsistent with this study, other studies revealed that there was an increased risk of premature delivery in mothers with PIH and also the perinatal deaths observed on eclamptic mothers were commonly caused by prematurity of the newborn(23, 24).

In line with the study conducted in Southern Ethiopia, Jewish and Kerman (14,25,26), the finding of this study revealed that the risk of perinatal death decreased as the number of ANC visits increased. This perinatal mortality might be due to the delay in recognition and initiation of interventions. The delay in providing treatment in the early stage of the disease is likely to progress to severe stage of the disease like severe preeclampsia and eclampsia, which was in turn found to have a strong association with perinatal mortality.

Similar with other studies, this finding revealed that mothers who gave birth through vaginal delivery had a higher risk of perinatal mortality compared to the counterparts by cesarean delivery. This might be due to cesarean delivery results in an expeditious delivery and also it has reduced risk of complications associated with PIH and perinatal mortality (14). In addition, the strong association of perinatal mortality with vaginal delivery may not necessarily show the increased risk because most of the still births occurred before the onset of labor or before CS was considered.

This study revealed that women with a preparum onset of PIH had higher risk of perinatal mortality. This is probably due to the early occurrence of PIH; it has a high opportunity to develop complications and to have eclampsia. Due to the severe nature of eclampsia, which potentially complicate the problem by severe intrauterine asphyxia, severe placental abruption and neonatal sepsis (27). In addition, the antepartum onset of PIH accounted for most of the perinatal deaths (91%) of deaths among mothers with pregnancy induced hypertension.

Mothers who had the highest systolic blood pressure of 160 and above had two folds increased risk of perinatal mortality. This might be due to the fact that complications associated with PIH were elicited when the range of highest blood pressure increased.

Similarly, this study did not indicate a statistically significant association of perinatal mortality with type of anticonvulsant given. Also, another study reported that there was no difference in perinatal mortality between diazepam and magnesium sulphate groups (14,28). Another study, however, showed that women with diazepam had 3 times higher risk of perinatal death compared to women with magnesium sulphate (29). Also, it is noted that magnesium sulphate is better in preventing and controlling convulsion in women presenting with PIH (30), which indirectly prevents perinatal death through controlling eclampsia. However, these inconsistent findings need meta-analysis.

The limitation of this study was that since the study was conducted through record review, some variables related with laboratory tests, socio-economic, socio-demographic and service related factors were missed. Similarly, the measurements for antepartum and post-partum care were not comprehensive, particularly in relation to referral and medication unavailability.

In conclusion, the multivariable analysis indicated that high parity, low gestational age, low number of ANC visits, low birth weight, vaginal delivery, antepartum onset of HDP and highest systolic blood pressure more than 160mmHg were the independent predictors of perinatal mortality. The government should highly encourage the pregnant mothers to reduce the number of births and educated on ANC visits to early determine PIH. In addition, the hospitals should give due attention to early determination and initiate treatment of PIH. On the other hand, follow up of mothers under PIH should be strengthened by the health professionals.
